# Chemical Account of the Angustura of Commerce, Wherein the Means Are Pointed out of Ascertaining the True Kind

**Published:** 1809-08

**Authors:** L. A. Planche

**Affiliations:** Chemist and Apothecary


### *Chemical Account of the Angustura of Commerce, wherein the Means are pointed out of ascertaining the true Kind.* By L. A. Planche, Chemist and Apothecary.*

THE remedy which forms the subject of this article has been hailed by some medical men as a febrifuge of equal efficacy with the best Peruvian bark; but whether from administering it under unfavourable circumstances, or from not having employed the true kind, other practitioners have disputed the febrifuge powers of Angustura. Hence arises the uncertainty which prevails, as to the efficacy of this remedy; hence, the necessity of trying new experiments in order to establish, in a more accurate manner than has hitherto been done, its medicinal properties. These experiments certainly belong to the practice of medicine; I shall therefore consider the question in this light.

The

---

* From Journal General de Medicine.

The following pages will therefore have for their object, 1. To detail some chemical and physical properties of several kinds of bark, sold by the name of Angustura. 2d. By comparing them together to ascertain some characteristics, by which we may distinguish them from each other. 3d. To call the attention of the practitioner, not only to the true Angustura, but also to new kinds of bark, long confounded with the first discovered, and the virtues of which are necessarily different. From this outline, it is evident, that I do not mean to confine myself to a chemical analysis, properly so called, but rather to give a hasty sketch, intended to clear the way for the physician who wishes to administer this remedy.

Under the name of Angustura, there are to be found in the shops of the Druggists, three kinds of barks, very distinct from each other, which I shall describe under the following names:

First species, *Angustura vera;* second species, *Pseudo Angustura, ferruginea, cortice convoluta*; third species, *Pseudo Angustura cortice plana.*

First species, *Angustura vera,* the oldest known, is that which has been described by Murray, in his Apparatus Medicaminum, and faithfully repeated by Dr. Alibert in his Materia Medica.

"This bark is a little convex, generally broader and thicker than cinchona. The epidermis covering it is whitish, unequal, and full of asperities; the substance covered by this epidermis is of a fawn coloured brown, and of a hard and firm texture.

"When reduced into powder, it has a very yellow appearance. I designate the first species in this manner, because it was in fact, the only one used in France at the time of the introduction of Angustura into medicine. I was the more scrupulous in ascertaining its quality, from regarding it as a test of comparison in my future experiments."

1st Species, *Angustura vera* and cold water.

Two drachms of Angustura being macerated twenty-four hours with eight ounces of cold water, in a temperature of about 10° above *Zero (Reaumur)* a clear liquor is obtained, which passes very speedily through the filtering paper; it is of the colour of table beer, of a nauseous smell, like that of briony; and is of a bitter aromatic taste.

a. This liquor gives a yellow precipitate with the sulphate of iron.

b. The b. The nitrate of silver forms a very abundant white precipitate, which in an hour becomes a more or less deep grey, at the place where it is most directly in contact with the light; soon afterwards, namely, in two hours the whole precipitate acquires a dirty purple colour.

c. The sulphate of copper is precipitated in it, in yellowish flakes, inclining to green.

d. Solution of isinglass does not present any remarkable phenomenon with the maceration of Angustura; the two mixed liquids keep clear a long time.

The bark of Angustura reduced to powder, and subjected to the action of very weak muriatic acid, communicates to this liquid the property of precipitating in a fine citron yellow, with the precipitate of potash.

### *Action of boiling Water upon Angustura in Powder.*

The filtered decoction of Angustura, deeper coloured than the foregoing liquor, is not perceptibly disturbed upon cooling. It acts almost in every respect, the same way with the small number of re-agents I have mentioned.

### Second Species, *Pseudo Angustura, ferruginea, cortice convoluta.*

This kind has not yet been described by any writer on the Materia Medica; it is at present very common, and several druggists describe it by the appellation of *fine Angustura.*

Messrs. Jussieu and Bompland, to whom I shewed it, declared that they were ignorant of the vegetable to which it belonged.

The barks of this species are generally rolled over each other, and are of a yellowish grey colour internally. Some of them have the epidermis covered with a substance which has the appearance of iron rust, and which possesses some properties. Other barks are more or less shining, sometimes very much wrinkled and studded with various coloured spots.

These last barks are generally thicker, and more voluminous than the others; and although they differ in appearance, they possess the same chemical properties; they are merely a little less ferruginous.

The powder of this false Angustura is of a grey colour, similar to ipecacuanha, and has a similar smell. It is so bitter that f[illegible] persons can taste it without experiencing nausea.

If this powder is macerated with water, in the same proportion, and during the same time with the true Angustura, a liquor a liquor is obtained, which when filtered, is of a straw colour, not sensibly altered by the contact of air, of a faint smell, and bitter, like pulverized bark, precipitating in a dark grey with sulphate of iron, and forming with the nitrate of silver, a white precipitate, which becomes perfectly black in five or six minutes. The sulphate of copper forms a precipitate less highly coloured, and less abundant than with the true Angustura; it is not affected by the solution of glue.

Lastly, Water sharpened with muriatic acid, and shaken with the powder of this false Angustura, assumes a clear green colour, if alkaline prussiate be poured into it; and soon afterwards Prussian blue is deposited. It is proper to remark, that we instantly obtain a very fine Prussian blue, if we treat the yellow powder which covers the bark with the muriatic acid; this evidently proves the above substance to be of a ferruginous nature, and is a phenomenon not observable with the true Angustura bark.

The decoction of ferruginous Angustura is higher coloured than its water of maceration; although transparent when warm, it becomes turbid on cooling, like cinchona. We may however avoid this inconvenience, if it be one, by adding a little solution of glue, which has the property of keeping the cooled liquor transparent. In some cases, we might derive great advantage from this method, both in correcting the extreme bitterness of the decoction, and diminishing the disgust which a turbid drink almost always creates in a patient. As to the metallic salts, such as the sulphates of iron, copper, and nitrate of silver, they act nearly in the same manner upon the water of maceration, and upon the decoction of the false ferruginous Angustura.

To conclude, this bark, which surpasses in bitterness all the bitter vegetables known, is well worthy the attention of medical practitioners.

Third Species, *Pseudo Angustura cortice plana*.

This third species is in small estimation among the druggists, who sell it at a very low price. At first sight it has some resemblance to the true Angustura; but on examining it more attentively, we perceive that it differs from it; 1st. in the internal colour of the bark, which is of a deep yellow, inclining to red; 2d. by its rougher and less resinous fracture; 3d. by its slightly bitter taste; 4th. by the colour of the powder, which resembles the grey cinchona, and some of the properties of which it possesses. I am of opinion opinion that this bark, which has no other name in commerce than that of common Angustura, is only a variety of the *Cinchona Magnifolia* of Bompland.

1st. The powder of the false *Angustura cortice, plana*, communicates a bright yellow colour to water, which becomes a reddish brown on exposure to the air.

2d. This water of maceration is more abundant in mucous principles than that of the two former barks, but although separated in the same manner, does not pass so easily through filtering paper; it unites in a feeble degree the smell and taste of the grey Kina of the shops.

3d. The solution of glue produces a very abundant and flaky precipitate.

4th. It gives with the sulphate of iron, a very dark green precipitate.

5th. With the nitrate of silver, a dirty grey precipitate, which keeps this colour a long time.

6th. With the sulphate of copper, some slight flakes of a greyish colour.

7th. The decoction of the false Angustura of this third species is of a very deep red colour; it is transparent both when hot and cold; it precipitates glue more abundantly than when macerated, and acts in every respect similar, both in decoction and maceration, with the reagents.

The information I have now communicated, is the result of some experiments tried about eighteen months ago for my own use, and which I communicated at the time to M. Jaquemin.

I ought to observe, however, that it is only within these two months I heard of the existence of a third kind of Angustura in commerce, and this part of my experiments is here.

In imitation of the example of M. Vauquelin, when treating of Cinchona, I have drawn up the subjoined table of the chemical phenomena observed, with respect to Angustura.

A Table

| Species. | Sulphate of Iron. | Nitrate of Silver. | Sulphate of copper. | Solution of Glue. | Weak Muriatic Acid and Prussiate of Potash. | Observations. |
|---|---|---|---|---|---|---|
| First. *Angustura Vera.* | Yellow precipitate. | Abundant white precipitate which became grey after an hour, and dirty purple in two hours. | Precipitate, in greenish yellow flakes. | 0 | Citron yellow; pulverised bark is here intended. | The first four columns indicate the effects produced upon the maceration and decoction. |
| Second. Pseudo-Angustura ferruginea cortice convoluta. | Dark grey precipitate. | White precipitate which became black in five or six minutes. | Thin apple green precipitate. | Hinders the decoction from being disturbed on cooling. | A fine green colour at first; afterwards a precipitate of Prussian blue. | There is an instantaneous production of Prussian blue, if we treat the yellow powder with the muriatic acid and an alkaline Prussiate. |
| Third. *Pseudo-Angustura cortice plana.* | Very dark green precipitate. | Dirty grey precipitate. | Dirty white flaky precipitate. | Very abundant flakes. | 0 | 0 |

